# Information and Risk Modification Trial (INFORM): design of a randomised controlled trial of communicating different types of information about coronary heart disease risk, alongside lifestyle advice, to achieve change in health-related behaviour

**DOI:** 10.1186/s12889-015-2192-5

**Published:** 2015-09-07

**Authors:** Barbora Silarova, Joanne Lucas, Adam S. Butterworth, Emanuele Di Angelantonio, Christine Girling, Kathryn Lawrence, Stuart Mackintosh, Carmel Moore, Rupert A. Payne, Stephen J. Sharp, Guy Shefer, Zoe Tolkien, Juliet Usher-Smith, Matthew Walker, John Danesh, Simon Griffin

**Affiliations:** MRC Epidemiology Unit, University of Cambridge, Institute of Metabolic Science, Cambridge, CB2 0QQ UK; Cardiovascular Epidemiology Unit, Department of Public Health and Primary Care, Strangeways Research Laboratory, Wort’s Causeway, Cambridge, CB1 8RN UK; The INTERVAL trial coordinating centre, Department of Public Health and Primary Care, Strangeways Research Laboratory, Wort’s Causeway, Cambridge, CB1 8RN UK; Patient and Public Involvement representatives, Cambridge, UK; Cambridge Centre for Health Services Research, University of Cambridge, Institute of Public Health, Forvie Site, Robinson Way, Cambridge, CB2 0SR UK; The Primary Care Unit, Department of Public Health and Primary Care, University of Cambridge, Strangeways Research Laboratory, 2 Wort’s Causeway, Cambridge, CB1 8RN UK

**Keywords:** Behaviour, Cardiovascular diseases, Communication, Genetic, Phenotypic, Physical activity, Protocol, Randomised controlled trial, Risk

## Abstract

**Background:**

Cardiovascular disease (CVD) remains the leading cause of death globally. Primary prevention of CVD requires cost-effective strategies to identify individuals at high risk in order to help target preventive interventions. An integral part of this approach is the use of CVD risk scores. Limitations in previous studies have prevented reliable inference about the potential advantages and the potential harms of using CVD risk scores as part of preventive strategies. We aim to evaluate short-term effects of providing different types of information about coronary heart disease (CHD) risk, alongside lifestyle advice, on health-related behaviours.

**Methods/Design:**

In a parallel-group, open randomised trial, we are allocating 932 male and female blood donors with no previous history of CVD aged 40–84 years in England to either no intervention (control group) or to one of three active intervention groups: i) lifestyle advice only; ii) lifestyle advice plus information on estimated 10-year CHD risk based on phenotypic characteristics; and iii) lifestyle advice plus information on estimated 10-year CHD risk based on phenotypic and genetic characteristics. The primary outcome is change in objectively measured physical activity. Secondary outcomes include: objectively measured dietary behaviours; cardiovascular risk factors; current medication and healthcare usage; perceived risk; cognitive evaluation of provision of CHD risk scores; and psychological outcomes. The follow-up assessment takes place 12 weeks after randomisation. The experiences, attitudes and concerns of a subset of participants will be also studied using individual interviews and focus groups.

**Discussion:**

The INFORM study has been designed to provide robust findings about the short-term effects of providing different types of information on estimated 10-year CHD risk and lifestyle advice on health-related behaviours.

**Trial registration:**

Current Controlled Trials ISRCTN17721237. Registered 12 January 2015.

**Electronic supplementary material:**

The online version of this article (doi:10.1186/s12889-015-2192-5) contains supplementary material, which is available to authorized users.

## Background

Cardiovascular disease (CVD) remains the leading cause of death globally [1], with an estimated 17 million deaths from CVD in 2011, or about 3 in every 10 deaths [2]. Of CVD deaths, 7 million deaths are due to coronary heart disease (CHD) and 6.2 million due to stroke [3]. The annual number of people who die from CVD is predicted to reach 23.3 million by 2030 and CVD is projected to remain the single leading cause of death [4].

Primary prevention strategies often involve CVD risk scores [5–9] to identify individuals at high estimated risk of CVD in order to target preventive interventions. Risk scores, which are recommended for use by national guidelines in most countries [10, 11], are algorithms that calculate an individual’s risk of CVD by combining information on several different risk factors. In the United Kingdom, CVD risk assessment tools are implemented in general practitioner (GP) computer systems [12]. The National Health Service (NHS) has initiated a programme of CVD risk reduction (“NHS Health Check”) which includes assessment of CVD risk for all those aged 40–74 years without pre-existing CVD and related disorders, although the effectiveness of this approach remains uncertain [13].

There are several risk factors that increase the risk of CVD including both non-modifiable factors such as family history, ethnic origin and gender and modifiable risk factors such as smoking, unhealthy diet, obesity and diabetes [14, 15]. The UK National Institute for Health and Care Excellence (NICE) [16] and the Joint British Societies for the prevention of CVD [10] recommend primary prevention of CVD through a focus on interventions that may change the behaviour of individuals, such as smoking cessation, reducing alcohol consumption, and increasing physical activity [17]. The provision of CVD risk information could afford the opportunity to improve CVD risk perception among individuals and so motivate a change in health-related behaviours. There is evidence to suggest that the provision of phenotypic CVD risk information improves people’s accuracy of perceived risk. Systematic reviews have, however, reported that provision of risk information needs to be accompanied by other tools in order to improve health-related behaviours and other relevant clinical outcomes [18, 19]. Studies included in previous reviews have been limited by the use of self-reported (rather than objective) measures of diet and physical activity.

Recent identification of several genetic risk factors for CVD has suggested the possibility of supplementing traditional CVD risk scores with genetic information to help motivate behaviour change and improve clinical outcomes [20, 21]. Theoretical models of health-related behaviour change, such as the Health Belief Model [22], would predict a positive impact of using such an approach. Direct-to-consumer genetics companies have claimed that providing people with such information could encourage healthy lifestyle choices. Alternatively, however, provision of such information could induce a defeatist response, leading to unhealthy lifestyle choices. A review by Marteau et al. [23] concluded that provision of genetic risk information generally has little or no effect on behaviour, although it may have a small effect on intentions to change behaviour. However, previous studies of the impact of provision of genetic risk information have been underpowered and lacked objective measures of behaviour. Hence, the potential benefits and the potential harms of adding, and communicating, genetic CVD risk information to phenotypic CVD scores are unknown.

Using both quantitative and qualitative methods, we aim to assess the short-term effect of providing different types of information about CHD risk, alongside lifestyle advice, on health-related behaviours.

### Objectives

#### Primary objective

The primary objective of the INFORM study is to evaluate the effect of providing different types of information about estimated 10-year CHD risk, alongside lifestyle advice on health-related behaviours over three months.

#### Secondary objectives

The secondary objectives are to evaluate the effect of providing different types of information about CHD risk, alongside lifestyle advice on objectively measured dietary behaviour (key secondary objective); cardiovascular risk factors; current medication and healthcare usage; perceived risk; cognitive evaluation of provision of CHD risk scores; and psychological outcomes.

#### Objectives of the qualitative study

The study also aims to provide rich and detailed information which may explain, clarify, support or modify the quantitative findings.

## Methods/Design

### Study design

**Fig. 1:**
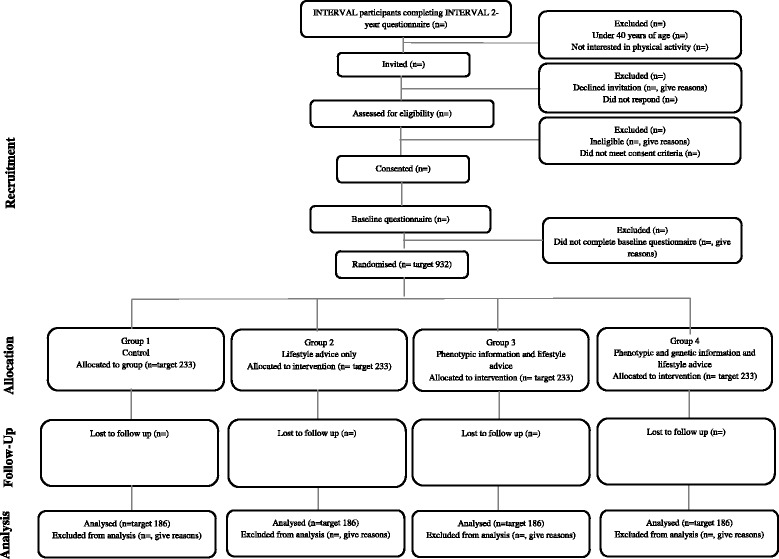
INFORM Trial CONSORT Diagram

The design of the trial and flow of participants are shown in Fig. 1. In a parallel-group, open randomised trial, we are allocating 932 male and female blood donors with no previous history of CVD aged 40–84 years in England to either no intervention (control group) or to one of three active intervention groups: i) lifestyle advice only ii) lifestyle advice plus information on estimated 10-year CHD risk based on phenotypic characteristics; and iii) lifestyle advice plus information on estimated 10-year CHD risk based on phenotypic and genetic characteristics. Lifestyle advice consists of three sessions of interactive, tailored web-based information. The qualitative element of the study consists of individual interviews and focus groups with trial participants at different time points throughout the trial.

### Population

Male and female blood donors aged 40–84 years with no previous history of CVD.

### Setting

The trial is administered at the University of Cambridge. Participants live in England.

### Recruitment

Participants are a subset of those recruited into the INTERVAL study, an ongoing randomised, multisite trial that has recruited approximately 50,000 blood donors from all 25 permanent donor centres of NHS Blood and Transplant (NHSBT) throughout England. The INTERVAL study aims to determine whether donation intervals can be safely and acceptably decreased to optimise blood supply while maintaining the health of donors [24]. Blood donors are potentially eligible to participate in the INTERVAL study if they are aged 18 years or older, fulfil all normal criteria for blood donation [25] and are willing to donate at one of the permanent NHSBT donation centres over a period of two years. Participants are excluded if they do not have internet access and/or are not willing to provide an email address for trial correspondence, since the trial mainly collects data via remote and web-based methods [24]. INTERVAL participants provide data (via online questionnaires) and blood samples at the time of joining the study and after two years. DNA has been extracted from baseline blood samples for genome-wide genotyping using the Affymetrix “UK Biobank” Axiom genotyping array. In addition, lipid profiles of all participants are being assessed via NMR-metabolomic assay. At their 2-year anniversary of joining INTERVAL, a subset of participants, who are willing and selected to do so, wear a physical activity monitoring device for 7 days (Axivity AX3 3-Axis Logging Accelerometer®, Axivity, York, UK).

INTERVAL participants are invited to take part in the INFORM study if they have completed their INTERVAL 2-year follow-up questionnaire and have indicated an interest in wearing a physical activity monitor. Furthermore, they have to be aged 40–84 years (inclusive); willing to provide a blood sample; have sufficient data available to the study team for calculation of phenotypic and genetic risk estimates for CHD (described in detail below); agree to allow trial staff to contact their GP to notify them of trial participation and study test results; have internet access and are willing to provide an email address for study correspondence; and have a good understanding of the English language, both written and oral (study materials are not tailored to support non-English language speakers). Potential participants are excluded if they have prior history of CVD (e.g. heart attack, angina, peripheral arterial disease or stroke, surgical or percutaneous coronary revascularisation procedure); a medical condition or disability that means the participant cannot engage in physical activity; known pregnancy at time of recruitment; are unable to provide informed consent; and are currently participating in another interventional clinical trial in cardiovascular risk or lifestyle modification (e.g. diet, physical activity or smoking cessation).

Between March 2015 and June 2015, electronic invitations approved by the local ethics committee have been sent to participants who meet the invitation criteria. The invitation email message consists of a brief summary and a personalised weblink to the INFORM screening and consent questionnaire. It also contains a weblink to the study website [26], where participant information is available.

We aim to involve a subset of 30–35 participants in the INFORM trial to take part in qualitative interviews and a further subset of 16–36 participants to take part in 2–3 focus groups. The exact number of the interviewees and focus groups is dependent on saturation of the data and the willingness of enough participants from the same area to take part in focus groups. Invitations to take part in the qualitative interviews and focus groups are sent to a purposive sample selected from participants who have given their consent to be approached about the qualitative study when completing the online consent form to take part in the trial. The invitation email message for the qualitative study consists of an invitation and participant information leaflet.

### Consent

Potential participants are providing implied consent for the INFORM Screening questionnaire by completing the questionnaire and those who are eligible progress to online informed consent for study entry. As part of the informed consent participants provide consent to provide a blood sample. When participants visit their general practice to have their blood sample taken, practice staff will take verbal consent for the procedure at the time of the visit and this will be recorded in the patient notes. The qualitative interview and focus group participants will be asked to sign a paper consent form before the start of the interview or focus group.

### Baseline assessment

Baseline information is collected prior to randomisation. Responders who are eligible to take part in the INFORM study and provide informed consent are sent a welcome email message with an individually tagged URL link to the baseline online questionnaire (available from the authors on request). Participants are requested to complete this questionnaire within 7 days and receive reminder email messages at day 7 and day 14. A reminder telephone call will be made on day 16 if the questionnaire still has not been completed. The measures assessed in the baseline questionnaire are combined with several measures taken during the participants’ INTERVAL study assessment in order to characterise the study population and to calculate individual’s phenotypic and genetic risk scores.

For consenting participants in INFORM who took part in physical activity monitoring in the INTERVAL study, physical activity data will be shared between studies and used as a baseline measurement for the INFORM study. Participants who did not take part in physical activity monitoring during the INTERVAL study are required to undertake baseline physical activity monitoring over 7 days. The study team sends participants a watch-like device (Axivity AX3 3-Axis Logging Accelerometer®) to measure physical activity over a period of 7 days. The device is sent together with instructions and a list of frequently asked questions and responses to the questions. A letter detailing the “switch on” and “switch off” date (pre-set by the study team before posting) is included with the device with instructions for return to the study team in the freepost envelope provided. Participants are reminded by email to return their monitor 6 days after the “switch off” date. If the monitor is not returned after the email message is received, the helpdesk will attempt to contact the participant by telephone or email. If a response is still not received, a written request for the return of the monitor will be sent after 5 weeks. If the monitor is still not returned after 6 weeks, the participant is randomised.

### Randomisation, allocation concealment and blinding

Randomisation of the INFORM participants is undertaken centrally at the trial coordinating centre at the Department of Public Health and Primary Care, University of Cambridge. The study involves individual-level randomisation using a computer program built into the study database. This programme is designed and implemented by the data manager. Randomisation is stratified by age (≤ or > 60 years) and sex in order to balance baseline phenotypic risk across study groups. Given the nature of the trial, it is not possible to blind participants to which intervention they receive. However, an independent data manager (not involved in designing the study) oversees the randomisation and data collection process. Additionally, the researchers assessing the trial outcomes will remain blinded to the allocation of interventions. As the participants for the qualitative part of the study are purposively selected, the qualitative researchers have access to the information collected during baseline assessment and treat it as confidential.

### Interventions

The study arms are summarised in Table 1. All participants will receive any information omitted from their group allocation at the end of the follow-up period (this includes the control group). The intervention in the INFORM study consists of three components provided to groups as described in Table 1: phenotypic CHD risk score; genetic CHD risk score; and lifestyle advice.

**Table 1:** Study Arms

Group	Type	Lifestyle advice	Phenotypic risk score	Genetic risk score
1	Control	No	No	No
2	Intervention	Yes	No	No
3	Intervention	Yes	Yes	No
4	Intervention	Yes	Yes	Yes

#### Phenotypic CHD risk score

The phenotypic CHD risk score consists of three pieces of information (Additional file 1A for example) as adapted from a study by Persell et al. [27]. First, we provide participants with information on their absolute risk of having CHD in the next 10 years, both as a percentage and as a natural frequency. We used the same set of traditional CVD risk factors (age, sex, smoking status, blood pressure, diabetes mellitus and total cholesterol and high-density lipoprotein – HDL – cholesterol) as used in many existing CVD risk scores [28] to calculate the absolute risk of having either fatal or nonfatal CHD events during the next 10 years. However, instead of objectively measured systolic blood pressure (as used in existing CVD risk scores), we use self-reported information on prescribed antihypertensive medication as a proxy for blood pressure (since it is not logistically feasible to collect objectively measured blood pressure in this study). We therefore refined an existing validated phenotypic CVD risk score [29] to take account of this change in variable. In line with D’Agostino et al. [29], we used sex-specific Cox proportional-hazards regressions to relate risk factors to the incidence of first CHD event. Correlates included in the Cox models were age, total cholesterol (mmol/l), HDL cholesterol (mmol/l), antihypertensive medication (self-reported yes/no), current smoking (self-reported yes/no) and diabetes mellitus (self-reported yes/no). All the continuous variables were log-transformed. From these models, we estimated mathematical CHD functions to predict 10-year risk of CHD, using the general formula as published by D’ Agostino et al. [29].

Second, the absolute risk of having CHD in the next 10 years is accompanied by “Heart Age”. An individual’s heart age is the chronological age of someone with the same absolute risk of CHD but with healthy risk factors. This means that individuals with elevated CHD risk factors will have a higher heart age than their chronological age. The definition of “normal” is based on the following profile: not a current smoker, does not have diabetes, not using antihypertensive medication, total serum cholesterol = 4.6548 mmol/l and HDL cholesterol = 1.1637 mmol/l [30]. The “Heart Age” was derived from the same general formula as used for the absolute risk score, rearranged such that age is unknown, other risk factors are normal (as defined above) and absolute risk is that of the participant in question.

Finally, we provide participants with a comparative risk estimate to encourage changes in health-related behaviours. An individual’s comparative risk is the risk of someone who is the same age and sex and has healthy lifestyle-related factors that are associated with CHD risk [31, 32]. Healthy lifestyle-related factors were defined as follows: a) current non-smokers (i.e. never smoked and former smokers) [33]; b) moderate levels of alcohol consumption (one or more units a week but not more than fourteen units a week; 1 unit = 8 grams) [33]; c) consumption of fruit and vegetables (more than 400 grams) [34]; d) consumption of fish (a portion size of 140 grams cooked weigh (20 grams/day) [35]; e) consumption of red meat (≤6 portions a week, equivalent to ≤500 grams of cooked weight or 71 grams per day [36]; f) physical activity (not inactive, at least half an hour of leisure-time activity a day) [33]; g) body mass index (BMI) <25 kg/m^2^.

All three pieces of information (absolute risk, “Heart Age” and comparative risk) provided as part of the phenotypic CHD risk score were modelled using the data from the EPIC-Norfolk study [37]. This means that we ran Cox proportional-hazards regressions as described above in EPIC-Norfolk to get the coefficients, but each INFORM participant’s values are plugged in to these equations to get their 10-year risk (Additional file 1B for example). As the outcome for modelling, we have chosen CHD instead of CVD, since the majority of genomic loci known to relate to CVD are associated with CHD (rather than stroke) and the study we used to estimate the coefficient for our genetic risk score (EPIC-CVD) only has CHD endpoints available currently. Given the fact that CHD is the most common type of CVD the results of INFORM study should be also applicable for CVD risk communication.

#### Genetic CHD risk score

As with the phenotypic CHD risk score, the genetic CHD risk information consists of three pieces of information (Additional file 1C for example). First, we provide participants with the information on their absolute risk of having CHD in the next 10 years. Second, information on “Heart Age” is provided. Lastly, we provide participants with a comparative risk estimate.

At the time of study design, there were 49 known genomic loci robustly associated with risk of CHD [38]. We selected lead variants at 46 of these regions that were included on the Affymetrix “UK BioBank” Axiom genotyping array being used on INTERVAL participants, as well as a customised version of the Illumina “Exomechip” array that has been run in the EPIC-CVD study [39], which was used to derive the risk estimates. EPIC-CVD is a case-cohort study comprising of ~10,000 incident CHD cases and a similar number of randomly selected comparators. Participants who have been genotyped in the INTERVAL study have a weighted genetic risk score (GRS) estimated for them based on their genotypes at each of the 46 relevant variants (Additional file 1D). This means that for each individual, the number of CHD risk alleles (0/1/2) carried at each single nucleotide polymorphism (SNP) was weighted by the strength (log of the odds ratio) of that SNP’s association with risk of disease (to account for the fact that not all variants have equal strengths of association) and then summed across SNPs. Weights used were drawn from the replication or combined estimates of association from the studies that originally discovered the variant associations [38, 40–42] . For estimation of the association between the GRS and incident CHD outcomes, the GRS was natural log-transformed (lnGRS). In line with the phenotypic risk score, we estimated mathematical CHD functions to predict 10-year risk of CHD using a similar general formula. For the absolute genetic risk score, this formula was plugged with the same baseline cumulative hazard as the absolute phenotypic risk score, individual lnGRS and the mean lnGRS. For the purpose of “Heart Age”, normal lnGRS was defined as the sex-specific 10th centile (<1.043 for males, <1.045 for females). For the purpose of comparative risk estimate, healthy lnGRS was defined as bottom 10 % of lnGRS (Additional file 1E for example).

#### Format of phenotypic and genetic risk

Both CHD risk scores are presented in the same format to ensure that the only experimental condition is the addition of information on the risk of CHD based on genetic variants (and not the different formats of presenting the CHD risk information). In designing the CHD risk scores we have taken into account the evidence regarding the most effective methods for communicating CHD risk estimates [30, 43–46]. Based on this evidence, INFORM participants are provided with: i) a graphical format in the shape of a thermometer using colours to distinguish risk (red, yellow, green) [47], ii) “Heart Age” tool and iii) comparative risk information [45, 47] (Additional file 1A and 1C for example). In addition to the CHD risk estimates, we also provide participants with an explanation of the factors included in the calculation of their CHD risk scores, how to interpret the thermometer and who to contact in case of any questions, as well as a section with the answers to frequently asked questions (adapted from a randomised trial of personal genomics for preventive cardiology [48]; and from a study by Persell et al. [27]) in line with previous evidence that participants expressed a need to understand the numbers and values presented to them [49].

#### Lifestyle advice

For the purpose of the INFORM study, we are using a web-based lifestyle intervention for CHD prevention based on an intervention that was originally developed for the Heart to Health study [50]. The lifestyle intervention consists of three sessions of interactive, tailored information on the web (up to three hours of interventional contact). In previous studies, offering patients a risk-reducing strategy was associated with increased adherence and reduced CHD risk [51]. Therefore, a library of over 100 web pages providing advice on physical activity, diet, smoking and medication are tailored according to participants’ selection of risk-reducing strategies (the web pages are available from the authors on request). Each session may last up to 60 min in duration depending on participants’ individual pace with sessions delivered at monthly intervals. The first session begins with content-specific education on diet, physical activity and smoking cessation. This session also includes tips on how to overcome self-identified barriers to risk reduction and the creation of steps toward self-identified actionable goals. Sessions 2 and 3 include similar content; a participant begins by reviewing their progress toward goals, continues with education and tips to overcome barriers and finishes with identification of new goals [50].

### Follow-up assessment

Follow-up assessments take place at 12 weeks after randomisation and include an online questionnaire, physical activity monitoring and blood sample collection. Not all of the measures in the baseline questionnaire are repeated at follow up (Table 2). The follow-up questionnaire (available from the authors on request) should take no more than 20 min to complete. Participants are requested to complete this follow-up questionnaire and will receive two reminder emails at day 7 and day 14 after sending the follow-up questionnaire if it has not been completed. If we do not hear from participants by day 24, we will mark the data as missing.

**Table 2:** INFORM measures at each stage of the study

	Measure or Instrument name	INTERVAL	INFORM baseline	INFORM follow-up	Mode of assessment	Note
Primary outcome	Physical Activity Level*	✓	✓	✓	accelerometers	using the Axivity AX3 – 3Axis logging Accelerometer®
Secondary outcomes	Traditional risk factors					
	Fruit and vegetables intake	✓		✓	carotenoids	
	Weight		✓	✓	self-report	as used in Godino et al. [56] and Knowles et al. [48]
	Cholesterol Panel (total, HDL and LDL cholesterol, triglycerides)	✓		✓	blood serum	
	Fructosamine	✓		✓	blood serum	
	Nutrition Behaviour (fruit, vegetables, whole grains, fish, red and processed meat)		✓	✓	self-report	one-item questions that reflect the present prevention guidelines on CVD [16, 10]
	Physical Activity Level		✓	✓	self-report	EPIC-Norfolk Physical Activity Questionnaire [60]
	Smoking Status		✓	✓	self-report	
	Alcohol Consumption		✓	✓	self-report	
	Current Medication and Healthcare Usage		✓	✓	self-report	adapted version of the Health Services Research Unit Aberdeen questionnaire [61]
	Perceived risk					
	Comparative and Absolute Cardiovascular Risk		✓	✓	self-report	adapted according to Diefenbach et al. [62] and used in other risk communication studies [48, 56]
	Cognitive evaluation of provision of coronary heart disease risk scores					
	Understanding of Risk Scores			✓	self-report	as used in another trial of risk communication [56]
	Perceived Accuracy of Risk Scores			✓	self-report	as used in other risk communication studies [48, 56]
	Acceptability of the Intervention			✓	self-report	in line with the Heart to Health study [50]
	Psychological outcomes					
	Stress		✓	✓	self-report	as used in the Randomized Trial of Personal Genomics for preventive cardiology [48]
	Mood		✓	✓	self-report	adapted from the Patient Health Questionnaire [63]
	Coronary Heart Disease-related Worry		✓	✓	self-report	an adaptation of the Cancer Related Worry Scale [64]
	Genetic Risk-related Worry/Anxiety		✓		self-report	as used by Knowles et al. [48]
Moderators and mediators	Sociodemographic characteristics					
	Sex, Age, Ethnicity	✓			self-report	
	Marital Status		✓		self-report	
	Socioeconomic Status (level of education, level of income, living area)		✓		self-report	adapted from the European Health Interview Survey; deprivation level of the area based on the Index of Multiple Deprivation, Department for Communities and Local Government
	Numeracy Skills		✓		self-report	3-item Schwartz scale [65]
	Family History of Coronary Heart Disease		✓		self-report	
	History of Genetic Testing		✓		self-report	
	History of Coronary Heart Disease Risk Assessment		✓		self-report	
	Self-rated Health		✓	✓	self-report	Ware et al. [66]
	Barriers to risk-reducing strategy		✓		self-report	in line with the Heart to Health study [50]
	Cognitive and emotional theory-based antecedents to behaviour change					
	Intentions		✓		self-report	adapted according to Ajzen [67] and used in previous similar research [56]
	Perception of Diet		✓		self-report	
	Perception of Physical Activity		✓		self-report	
	Coronary heart disease risk Representations		✓		self-report	the Brief Illness Perceptions Questionnaire [68]
	Self-efficacy		✓		self-report	as used in previous behavioural research [56, 68]
	Response Efficacy		✓	✓	self-report	as used in previous behavioural research [52, 69]
	Social Support		✓		self-report	the Multidimensional Scale of Perceived Social Support [70]
	Time Orientation		✓		self-report	as used in a study by Peretti-Watel et al. [71]
	Sense of Coherence		✓		self-report	3-items scale, Lundberg et al. [72]
	Comparative Optimism		✓		self-report	comparative perceived risk

*participants who did not take part in physical activity monitoring during the INTERVAL Study will be required to undertake baseline activity monitoring over 7 days

#### Follow-up physical activity monitoring

All participants are requested to complete physical activity monitoring for 7 days at the 12-week time point following the same procedure as at baseline.

#### Blood sample

All participants are asked to provide a blood sample, which is taken at their general practice. The blood sample is used to provide information on participants’ lipid panel (total, HDL and low-density lipoprotein – LDL – cholesterol; triglycerides), carotenoid levels and fructosamine level. The study coordination team provides a blood sample kit (Royal Mail Safebox™) which is sent to participants along with the follow-up physical activity monitor. The blood sample kit includes blood collection tubes with barcoded, anonymised participant study number, instructions for general practice clinicians, general practice invoicing instructions and appropriate packaging addressed to the receiving central laboratory with pre-paid postage. A staff member at the participant’s general practice takes verbal consent for the procedure at the time it is taken, writes the date and time that the sample was taken on the blood collection tube, packs the sample, and enters it into the postal delivery chain alongside regular practice post. Samples are transported by Royal Mail first class mail to a central laboratory (UK BioCentre, Stockport, Cheshire, UK) that logs and processes the samples. An email reminder message to have a blood sample taken is sent 10 days after the sample kit has been posted. The helpdesk attempts to contact participants with outstanding blood samples on days 17 and 28 after the sample kit has been posted, and if the sample is not received after 31 days after the sample kit has been posted, blood sample results will be marked as missing data.

### Measures

Table 2 provides details of the measures collected in the INFORM study and the stage of the study at which each is assessed. The primary outcome is objectively measured physical activity, defined as average acceleration (m/s^2^) over the observation period. Participants are instructed to continuously wear the accelerometer for 7 consecutive days and nights, and to carry on with all normal activities during this time. The accelerometers are waterproof and can be worn while swimming and showering. Accelerometers have been chosen as a measure of physical activity because they cause low participant burden and are not subject to the reporting bias or recall problems associated with the self-report methods [52].

The key secondary outcome, dietary behaviour, is objectively measured by levels of six serum carotenoids (alpha-carotene, beta-carotene, lutein, lycopene, beta-cryptoxanthin, zeaxanthin). Carotenoids are biologically active pigments in plants but are not synthesised in animals and as such they represent a valid biomarker of vegetable and fruit intake [53].

Other objectively measured secondary outcomes include total, HDL and LDL cholesterol and fructosamine. We also collect self-reported secondary outcomes via an online questionnaire. They include cardiovascular risk factors (self-reported weight, smoking status, alcohol consumption, physical activity and dietary behaviour); current medication and healthcare usage; perceived risk (perception of comparative and absolute risk); cognitive evaluation of provision of CHD risk scores (understanding, accuracy, acceptability); and psychological outcomes (anxiety associated with testing, fatalism, depression, stress and mood).

As with any other intervention, it is important to understand the mechanisms that explain why the provision of CVD risk information leads to change or not, and whether there are people with certain characteristics who may benefit more or less from CVD risk communication. To understand this, the following potential moderators and mediators of the relationship between provision of risk information and behaviour change are being measured as part of the INFORM study: a) sociodemographic characteristics (ethnicity, socioeconomic status, education, income, numeracy skills and marital status); b) history of genetic testing and CHD risk assessment; c) self-rated health; d) cognitive and emotional theory-based antecedents to behaviour change (behavioural intentions, perception of diet and physical activity, beliefs about CVD, self-efficacy and response efficacy, social support, mood and personality characteristics); and f) personal barriers to risk reducing strategies.

### Qualitative data collection

#### Qualitative interviews

An experienced qualitative researcher conducts in-depth individual interviews with a sub-sample of trial participants starting soon after the randomisation and finishing around 2–3 months after the 3-month follow-up period is complete. Interviews cover issues related to the interviewees’ experience of taking part in the trial; more specifically, their understanding of CHD risk, factors affecting their understanding, reaction to receiving information about risk and lifestyle advice, the extent to which understanding of risk contributes to motivation for behaviour change, the implementation of intention to change after receiving risk information and lifestyle advice, and the facilitators and barriers for such a change. The qualitative study focuses mainly on participants in the intervention arms because the lack of an intervention leaves little to discuss and explore qualitatively with the members of the control arm. However, in order to explore the impact of receiving different types of information in different stages, we aim to have up to six interviews with members of arms who initially received no intervention/only lifestyle advice/only phenotypic risk score, after they receive all their risk scores at the end of the intervention.

In order to supplement the quantitative data which is collected at only two time points (at baseline and 12 weeks later), we aim to interview different participants at different time points, interviewing some immediately after the randomisation, others half way through the intervention and others towards the end of the intervention or up to 3 months after it ends. Proportionally more participants whose risk score is medium or high are purposively recruited as this is the population most likely to have a stronger motivation to change their lifestyle. A subset of this group may be interviewed more than once – for example immediately after the start of intervention and then after the completion of the intervention in order to explore early expectations/motivation and compare them with later perceptions or behaviour. For the participants who do not express an intention to change their lifestyle and those who are approached at later stages of the intervention, the discussion on the reaction to receiving the risk scores or lifestyle advice is conducted retrospectively focusing on the period from receiving the risk scores and up to the point of the interview. Interviewees are asked to sign an interview consent form giving permission for their interview to be recorded.

#### Qualitative focus groups

Two to three focus groups are conducted at different points of time throughout the trial. Approximately 8–12 participants take part in each of the focus groups. These participants do not take part in the individual qualitative interviews. The focus groups are facilitated by an experienced qualitative researcher and one or two assistants from the research team. Participants are purposively selected in order to include people with various demographic backgrounds. However, at least two of the groups may be selected for gender homogeneity (male/female-only) in order to encourage greater participation in what may be considered a gender-sensitive issue. The focus groups cover issues related to the relationship between risk awareness, motivation for change and actual change of behaviour and lifestyle, but their main focus is on communication and understanding of CHD risk estimates as well as the lifestyle advice. We hope to benefit from the interactive aspect of the focus groups in order to gain as rich as possible feedback regarding the advantages and disadvantages of the forms of communication of risk and lifestyle advice used in the trial. Permission is sought from the participants, as outlined in the consent process, for the focus groups to be recorded and transcribed.

### Withdrawal

A participant is free to withdraw their consent from the study, at any time, without giving a reason. All requests from participants to withdraw from the study are directed to the study coordination team who will discuss the options for withdrawal. The study data manager places a flag on the research database to ensure appropriate use of data and notifies the study coordination team of withdrawal. University of Cambridge research staff instructs the central laboratory to remove all archive samples from the biorepository for participants who have been withdrawn from the study and who have requested no further use of their stored samples.

### Adverse events

This is a low-risk trial with little reason to consider that adverse events would arise as a result of following any of the interventions. Accordingly, no formal adverse event monitoring is planned. However, events may incidentally come to the attention of the research team (e.g. via interviews or focus groups, or direct communication with participants via the study coordination team) which will be recorded and followed up accordingly.

### Statistical analyses

#### Sample size calculation – randomised controlled trial

The primary outcome is between-group differences in the change (follow-up minus baseline) of the average acceleration (m/s^2^) over the observation period, measured using the Axivity AX3 3-Axis Logging Accelerometer®. The intervention will be potentially important if it increases the primary outcome by 10 %. This corresponds to an additional 10 min of brisk walking per day or an additional 3 min of jogging per day. The same effect size was used in the Understanding Risk trial by Price et al. [54] among people at high risk of CVD with one month of follow-up. This effect size is also in line with the ProActive trial that aimed to increase physical activity among individuals at high risk of type 2 diabetes [55]. To allow for four pairwise comparisons in the primary analysis, the significance level was set to 1.25 %. We calculated that 186 participants per group will be needed to detect this effect with 80 % power and 95 % confidence, assuming the estimated standard deviation (SD) of change in physical activity from baseline to follow-up is 0.05 and the correlation between physical activity at baseline and follow-up is 0.6 [56]. Allowing for an attrition rate of 20 %, we therefore aim to randomise a total of 932 participants (233 per group).

#### Sample size – qualitative study

A sample size of between 30 and 35 male and female participants will be selected purposively, mainly from the intervention arms to include people with various demographic backgrounds. This sample size is common for this kind of study [57, 58] and is consistent with the time resources required for this type of data analysis and the predicted range of number of interviews required for achieving saturation.

#### Analysis design – randomised controlled trial

All trial analyses will be based on the Intention To Treat (ITT) principle. Those with missing follow-up data will be excluded from the analyses (a complete case approach). We will perform sensitivity analyses to assess the impact of missing follow-up data on the results. Participants with missing baseline data will be included in the analyses using the missing-indicator method [59].

The null hypothesis for the primary analysis is that there is no difference between any of Groups 1 to 4. Estimates of the following pairwise differences will be calculated: 1) Group 1 (control group) vs. Group 2 (lifestyle advice only) – to estimate the effect of providing lifestyle advice compared with no intervention; 2) Group 3 (phenotypic risk score and lifestyle advice) vs. Group 4 (phenotypic and genetic risk scores and lifestyle advice) – to estimate the effect of providing a genetic risk score in addition to lifestyle advice and a phenotypic risk score; 3) Group 3 + Group 4 vs. Group 1 – to estimate the effect of providing risk score information and lifestyle advice compared with no intervention, and 4) Group 3 + Group 4 vs. Group 2 – to estimate the effect of providing risk score information in addition to lifestyle advice.

Mean changes in objectively measured physical activity between baseline and 3-month follow-up will be analysed using analysis of covariance (ANCOVA). The ANCOVA model will model change (follow-up minus baseline) in objectively measured physical activity, adjusted for baseline. A test involving three degrees of freedom (3 d.f.) will be performed of the null hypothesis that there is no difference between the four randomised groups. If the p-value from this test is <0.01, this will imply that there are differences between the groups. The ANCOVA model will also be used to derive estimates of the differences in mean change and 95 % confidence intervals for each of the four pairwise comparisons described above. To avoid detracting the focus of the results from the overall 3 d.f. test, *p*-values for each of the pairwise comparisons will not be calculated.

For each continuous secondary endpoint, the four pairwise differences will be estimated, together with 95 % confidence intervals, using ANCOVA as described for the primary outcome analyses (except Coronary Heart Disease-related Worry). The Coronary Heart Disease-related Worry will be measured only at follow-up, so the four pairwise differences will be estimated, together with 95 % confidence intervals, using linear regression. A 3 d.f. test will be performed of the null hypothesis that there is no difference between the four randomised groups. For categorical endpoints, similar analyses will be performed using logistic and multinomial regression models.

#### Subgroup analyses

For the primary endpoint, the ANCOVA model will be extended to include an interaction between the intervention effect and (1) age (continuous variable), (2) baseline phenotypic CHD risk score (continuous variable) and (3) sex. If the p-value for interaction is <0.05, then the number and percentage of individuals with the endpoint within each randomised group, together with intervention effect (and 95 % confidence intervals) will be reported separately within categories defined by the subgroup variable. For age and baseline CHD risk score, the cut-off for the categories will be their median values at baseline.

#### Multiplicity

Given the number of endpoints, randomised groups and therefore comparisons, the results for the primary endpoint will be regarded as convincing if the *p*-value from the 3 d.f. test is <0.01, while the results for each secondary endpoint will be regarded as convincing if the relevant *p*-value from the 3 d.f. test is <0.001.

#### Qualitative analyses

Thematic analysis will be used to analyse the qualitative data. The transcriptions will be coded using NVivo software. To increase the rigour and validity of the analysis, and as a form of triangulation, the framework development (coding tree) will be conducted by a subgroup of three members of the team (the lead qualitative researcher and two others). In addition, a subset of 20 % of transcripts will be double-coded by two members of the research team, and disagreements will be discussed in order to reach consensus about interpretation and indexing. Analysis will involve two stages:

1. Data management which will include: a) familiarisation with the data, including reading transcripts and notes and/or listening to the audio dialogue in order to extract main themes and ideas; b) thematic framework development, identifying the key issues and concepts present in the data and creating a coding tree which will be conducted both inductively, based on the data, and deductively, based on the research questions; c) indexing the data.
2. Interpretation stage which will include focused or axial coding, defining the main concepts and mapping the ways in which different parts of the data are related to each other.

## Discussion

The INFORM randomised trial has been designed to contribute toward the understanding of the impact of provision of phenotypic and genetic CHD risk information on health-related behaviour change and other important clinical outcomes. Comparison between randomised trial arms will enable estimation of: 1) the effect of providing web-based lifestyle advice; 2) the effect of providing a genetic risk score in addition to lifestyle advice and a phenotypic risk score; 3) the effect of providing risk score information and lifestyle advice and 4) the effect of providing risk score information in addition to lifestyle advice. The INFORM study will also provide information on potential moderators and mediators between the intervention and health-related behaviour change and other clinical outcomes.

Even though the routine use of CVD risk scores is strongly advocated by most cardiovascular guidelines [10, 11], relatively little is reliably known about the benefits of the provision of different forms of CVD risk scores to individuals and about any potential harms (such as the possibility of increased anxiety or false reassurance). Previous systematic reviews [18, 19] have concluded that no robust recommendations can be made about the value of providing CVD risk information owing to limitations in previous studies, including lack of adequate power and lack of objective measures of health-related behaviours.

The INFORM study is well-placed to advance current knowledge on this topic because it is a trial that combines several advantages, including: a) power to identify clinically important differences between groups in this study’s principal outcomes; b) objective measures of health-related behaviours; c) simplicity of the interventions and relevance to primary care. If the intervention(s) in this trial prove to be effective, then the components of the intervention should be relatively easy to implement in primary care practice. Hence, the INFORM trial should provide a reliable evaluation of the impact of the provision of CVD risk information, thereby potentially informing policy decisions and guiding clinical practice about the routine use of CVD risk scores in primary care.

### Research governance

#### Ethical approval

Ethical approval was received from NRES Committee East of England – Cambridge Central (14/EE/1164) on 03/12/2014. NHS Research and Development assurance has been received by our lead CCG (Cambridgeshire and Peterborough CCG) and CRN: Eastern. The trial was prospectively registered at the ISRCTN Registry (ISRCTN17721237) on 12/01/ 2015. This paper details version 1.2 of the trial protocol dated 13/01/2015. The trial has been adopted by the NIHR Portfolio.

### Study sponsor

The University of Cambridge is the sponsor of this trial.

### Data handling and quality assurance

A Data Monitoring Committee is not considered appropriate for this trial considering that the likely risks to participants are known to be minimal, recruitment and follow-up are over a short period of time, and the data for this trial is both collected and stored online and, as such, is expected to have a minimal error rate. Any incidental safety information will be reported to the Trial Steering Committee (TSC). The Data Manager is responsible for the quality and integrity of data and will provide a data report to each TSC meeting. Data are stored in accordance with the University of Cambridge School of Clinical Medicine Information Governance policy which relates to sensitive/identifiable personal information collected and stored for the purposes of research.

### Patient and public involvement (PPI)

Two representatives from the PPI Panel at Cambridge University Hospitals NHS Foundation Trust were recruited in June 2014. PPI representatives have played an integral role in the INFORM study and contributed to the different stages of the research. More specifically, PPI representatives commented on the protocol, questionnaires, wording and format of the presentation of the risk score estimates, critically revised patient information sheets (both qualitative and quantitative) as well as the web-based lifestyle intervention. PPI representatives have a central role in the TSC. We expect that they will also be involved with developing newsletters and taking part in dissemination activities.

### Trial oversight

In addition to the Research Ethics Committee, the INFORM study is overseen by the TSC and Trial Management Group (TMG). The purpose of the TSC is to provide oversight of the clinical trial, with particular reference to adherence to the protocol, compliance with the Department of Health Research Governance Framework and the Guidelines for Good Clinical Practice, and the rights, safety and wellbeing of the trial participants. In addition to members of the research team, the Committee contains a number of independent members, specifically the Independent Chair, Independent Academics and PPI representatives. The purpose of the TMG is to monitor the objectives and progress of the trial, oversee day-to-day management of the study, consider any new information that may be relevant to the trial and discuss any scientific matters contained in, or relevant to, the trial. It includes investigators, the trial manager and project administrative assistant.

### Research dissemination and data preservation for sharing

The investigators will analyse data according to a predefined analysis plan in a timely manner. Members of the research team will be involved in reviewing drafts of the manuscripts, abstracts, and any other publications arising from the trial. The Chief Investigator will have final approval on all publications and press releases. Authorship of publications will be determined by ICMJE guidelines. A lay summary of the research findings will be available on the study website [29] and study participants will be notified by email when they are available.

The database manager will take responsibility for data curation and archiving, and all data sets will be kept securely with no access from unauthorised personnel. Data will be stored so that it can be accessed, used and understood by subsequent users. On completion of the data collection we will develop a data dictionary so that scientists can request release of a study dataset from the investigators. When the investigators have completed their planned analyses, the anonymised data will be made available for use by others and will be shared under appropriate data sharing agreements. Primary data and the Trial Master File will be retained securely in their original form for a minimum of 10 years.

### Trial status

Ongoing. Recruitment was completed in June 2015.

## Additional file

Additional file 1:
**Appendix A.** An example of presentation of phenotypic coronary heart disease risk score. **Appendix B**. Mathematical coronary heart disease functions to predict 10-year risk of coronary heart disease. **Appendix C**. An example of presentation of genetic coronary heart disease risk score. **Appendix D.** INFORM SNPs for genetic risk score. **Appendix E**. Mathematical formulas for calculation of risk estimates based on genetic risk score (GRS). (ZIP 112 kb)

## Abbreviations

ANCOVA: analysis of covariance
BMI: body mass index
CHD: coronary heart disease
CVD: cardiovascular disease
GP: general practitioner
GRS: genetic risk score
lnGRS: natural log-transformed genetic risk score
HDL: high-density lipoprotein
ITT: Intention to Treat principle
LDL: low-density lipoprotein
NHS: the National Health Service
NHSBT: NHS Blood and Transplant
NICE: National Institute for Health and Care Excellence
PPI: Patient and public involvement
SD: standard deviation
SNP: single nucleotide polymorphism
TMG: Trial Management Group
TSC: Trial Steering Committee
3d.f: 3 degrees of freedom test
