# Supplementary material for: Information and Risk Modification Trial (INFORM): design of a randomised controlled trial of communicating different types of information about coronary heart disease risk, alongside lifestyle advice, to achieve change in health-related behaviour
Source: BMC Public Health. 2015 Sep 7;15:868. doi: 10.1186/s12889-015-2192-5 (PMC4562192; doi:10.1186/s12889-015-2192-5)
Supplement: Additional file 1: — Appendix A. An example of presentation of phenotypic coronary heart disease risk score. Appendix B. Mathematical coronary heart disease functions to predict 10-year risk of coronary heart disease. Appendix C. An example of presentation of genetic coronary heart disease risk score. Appendix D. INFORM SNPs for genetic risk score. Appendix E. Mathematical formulas for calculation of risk estimates based on genetic risk score (GRS). (ZIP 112 kb) [file 12889_2015_2192_MOESM1_ESM.zip › Additional file 1C.docx]

**Additional file 1C.** An example of presentation of genetic coronary heart disease risk score.

**Your risk of coronary heart disease based on genetic factors**

**
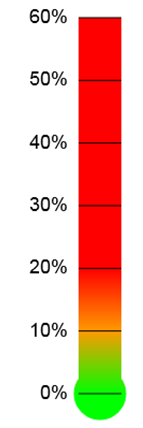
**

Risk for a woman with an optimal genetic risk profile

15%

Your risk

31%

**Your risk now**

Your risk of having coronary heart disease during the next 10 years is 31%. This means that approximately 31 out of 100 women like you (31%) will experience coronary heart disease in the next 10 years.

**Your Heart Age**

Your heart age is 83. This means that your risk of having coronary heart disease in the next 10 years is about the same as an 83-year-old woman who has an optimal genetic risk profile associated with coronary heart disease.

**For comparison,** a woman the same age as you with an optimal genetic risk profile has a 15% risk of having coronary heart disease during the next 10 years.

**Remember:**

- A high-risk estimate does not mean that you are destined to have coronary heart disease, and a low-risk estimate does not mean that you are completely without risk of having coronary heart disease.
- The genetic and coronary heart disease risk estimates are based on different factors, providing you with different information about your coronary heart disease risk. Do not attempt to combine them together to estimate your “total coronary heart disease risk” since this will not be an accurate estimate.
- No matter what the results are from the genetic risk estimate, a key aspect of good heart health remains – follow the advice we have given you regarding improving your diet, doing more exercise and giving up smoking (if applicable).

If you would like to know more about the genetic risk estimates please click on the following link (Frequently asked questions):

**Frequently asked questions**

**How many genes are involved in causing a coronary heart disease?**

For the vast majority of people, we believe that the risk of having coronary heart disease is influenced by many genes (probably hundreds) rather than just one or two.

**What does optimal genetic risk profile mean?**

The optimal genetic risk profile is defined as having less than 10% of the genetic characteristics associated with coronary heart disease.

**How important is family history of coronary heart disease?**

While family history is valuable information, family history not only includes your genetic risk but also your parents’ or siblings’ lifestyle choices, like whether they smoked or not. Based on recent developments in genetics, we are now able to estimate people’s risk much better using genetic information.

NOTE: The answers to frequently asked questions regarding risk estimates are adapted from a randomised trial of personal genomics for preventive cardiology by Knowles et al. 2012: *Randomized trial of personal genomics for preventive cardiology: design and challenges*; and from a study by Persell et al. 2013: *Electronic health record-based patient identification and individualized mailed outreach for primary cardiovascular disease prevention: a cluster randomized trial*.
