# Supplementary material for: Information and Risk Modification Trial (INFORM): design of a randomised controlled trial of communicating different types of information about coronary heart disease risk, alongside lifestyle advice, to achieve change in health-related behaviour
Source: BMC Public Health. 2015 Sep 7;15:868. doi: 10.1186/s12889-015-2192-5 (PMC4562192; doi:10.1186/s12889-015-2192-5)
Supplement: Additional file 1: — Appendix A. An example of presentation of phenotypic coronary heart disease risk score. Appendix B. Mathematical coronary heart disease functions to predict 10-year risk of coronary heart disease. Appendix C. An example of presentation of genetic coronary heart disease risk score. Appendix D. INFORM SNPs for genetic risk score. Appendix E. Mathematical formulas for calculation of risk estimates based on genetic risk score (GRS). (ZIP 112 kb) [file 12889_2015_2192_MOESM1_ESM.zip › Additional file 1D.docx]

**Additional file 1D.** INFORM SNPs for genetic risk score.

| ***Locus*** | **rsID** | **Odds ratio** | **Risk allele** | **Source** |
| --- | --- | --- | --- | --- |
|  |  |  |  |  |
| *SH2B3* | rs3184504 | 1.07 | T | CARDIoGRAMplusC4D |
| *ADAMTS7* | rs3825807 | 1.08 | A | CARDIoGRAM |
| *TCF21* | rs12190287 | 1.07 | T | CARDIoGRAMplusC4D |
| *LPA* | rs3798220 | 1.28 | C | CARDIoGRAMplusC4D |
| *ZC3HC1* | rs11556924 | 1.09 | C | CARDIoGRAMplusC4D |
| *ANRIL/CDKN2BAS* | rs10757278 | 1.23 | G | CARDIoGRAMplusC4D |
| *KCNK5* | rs10947789 | 1.06 | T | CARDIoGRAMplusC4D |
| *BCAP29* | rs10953541 | 1.08 | C | C4D |
| *PCSK9* | rs11206510 | 1.06 | T | CARDIoGRAMplusC4D |
| *LDLR* | rs1122608 | 1.10 | G | CARDIoGRAMplusC4D |
| *ANKS1A* | rs12205331 | 1.04 | C | CARDIoGRAMplusC4D |
| *CYP17A1, CNNM2, NT5C2* | rs12413409 | 1.10 | G | CARDIoGRAMplusC4D |
| *RASD1, SMCR3, PEMT* | rs12936587 | 1.06 | G | CARDIoGRAMplusC4D |
| *UBE2Z, GIP, ATP5G1, SNF8* | rs15563 | 1.04 | G | CARDIoGRAMplusC4D |
| *VAMP8-GGCX* | rs1561198 | 1.05 | T | CARDIoGRAMplusC4D |
| *PPAP2B* | rs17114036 | 1.11 | A | CARDIoGRAMplusC4D |
| *MIA3* | rs17465637 | 1.14 | C | CARDIoGRAM |
| *FURIN, FES* | rs17514846 | 1.05 | A | CARDIoGRAMplusC4D |
| *HDAC9* | rs2023938 | 1.07 | C | CARDIoGRAMplusC4D |
| *APOE* | rs2075650 | 1.11 | G | CARDIoGRAMplusC4D |
| *ZEB2-AC074093.1* | rs2252641 | 1.04 | C | CARDIoGRAMplusC4D |
| *SMG6, SRR* | rs2281727 | 1.05 | G | CARDIoGRAMplusC4D |
| *KIAA1462* | rs2505083 | 1.06 | C | CARDIoGRAMplusC4D |
| *LPL* | rs264 | 1.05 | G | CARDIoGRAMplusC4D |
| *SLC22A4-SLC22A5* | rs273909 | 1.09 | G | CARDIoGRAMplusC4D |
| *HHIPL1* | rs2895811 | 1.06 | C | CARDIoGRAMplusC4D |
| *COL4A1, COL4A2* | rs4773144 | 1.07 | G | CARDIoGRAMplusC4D |
| *IL6R* | rs4845625 | 1.04 | T | CARDIoGRAMplusC4D |
| *CXCL12* | rs501120 | 1.07 | T | CARDIoGRAMplusC4D |
| *APOB* | rs515135 | 1.08 | C | CARDIoGRAMplusC4D |
| *ABO* | rs579459 | 1.07 | C | CARDIoGRAMplusC4D |
| *SORT1* | rs602633 | 1.12 | G | CARDIoGRAMplusC4D |
| *ABCG5, ABCG8* | rs6544713 | 1.06 | T | CARDIoGRAMplusC4D |
| *FLT1* | rs9319428 | 1.05 | A | CARDIoGRAMplusC4D |
| *PHACTR1* | rs9369640 | 1.09 | A | CARDIoGRAMplusC4D |
| *PDGFD* | rs974819 | 1.07 | T | CARDIoGRAMplusC4D |
| *MRAS* | rs9818870 | 1.07 | T | CARDIoGRAMplusC4D |
| *MRPS6/KCNE2* | rs9982601 | 1.13 | T | CARDIoGRAMplusC4D |
| *LPA* | rs10455872 | 1.45 | G | IBC |
| *EDNRA* | rs1878406 | 1.06 | T | CARDIoGRAMplusC4D |
| *LIPA* | rs2246833 | 1.06 | T | CARDIoGRAMplusC4D |
| *GUCY1A3* | rs3796587 | 1.06 | C | CARDIoGRAMplusC4D |
| *PLG* | rs4252125 | 1.06 | G | CARDIoGRAMplusC4D |
| *ZNF259, APOA5-A4-C3-A1* | rs662799 | 1.11 | G | IBC 50K CAD Consortium |
| *WDR12* | rs6725887 | 1.12 | C | CARDIoGRAMplusC4D |
| *TRIB1* | rs6982636 | 1.04 | G | CARDIoGRAMplusC4D |

SOURCE:

CARDIoGRAMplusC4D Consortium, Deloukas P, Kanoni S, Willenborg C, Farrall M, Assimes TL. Large-scale association analysis identifies new risk loci for coronary artery disease. Nat Genet. 2013;45(1):25-33.

Schunkert H, König IR, Kathiresan S, Reilly MP, Assimes TL, Holm H, et al. Large-scale association analysis identifies 13 new susceptibility loci for coronary artery disease. Nat Genet. 2011;43(4):333-8.

IBC 50K CAD Consortium. Large-scale gene-centric analysis identifies novel variants for coronary artery disease. PLoS Genet. 2011;7(9):e1002260.

Coronary Artery Disease (C4D) Genetics Consortium. A genome-wide association study in Europeans and South Asians identifies five new loci for coronary artery disease. Nat Genet. 2011;43(4):339-44.
