# Supplementary material for: Information and Risk Modification Trial (INFORM): design of a randomised controlled trial of communicating different types of information about coronary heart disease risk, alongside lifestyle advice, to achieve change in health-related behaviour
Source: BMC Public Health. 2015 Sep 7;15:868. doi: 10.1186/s12889-015-2192-5 (PMC4562192; doi:10.1186/s12889-015-2192-5)
Supplement: Additional file 1: — Appendix A. An example of presentation of phenotypic coronary heart disease risk score. Appendix B. Mathematical coronary heart disease functions to predict 10-year risk of coronary heart disease. Appendix C. An example of presentation of genetic coronary heart disease risk score. Appendix D. INFORM SNPs for genetic risk score. Appendix E. Mathematical formulas for calculation of risk estimates based on genetic risk score (GRS). (ZIP 112 kb) [file 12889_2015_2192_MOESM1_ESM.zip › Additional file 1E.docx]

**Additional file 1E. Mathematical formulas for calculation of risk estimates based on genetic risk score (GRS)**

**Absolute genetic risk score**

- Men: P = 1 – 0.886^exp(ln(4.06)*ln(GRS) + ln(80.2)*ln(age) – ln(4.06)*1.192^ ^–^ ^ln(80.2)*4.14)^
- Women: P = 1 – 0.961^exp(ln(7.57)*ln(GRS) + ln(420.2)*ln(age) – ln(7.57)*1.189 – ln(420.2)*4.13)^

**Genetic Heart Age**

- Male heart age = exp^((ln((ln(1–P)/ln(0.886)) – K.man)/ln(80.2))^

where K.man= ln(4.06)*1.043 – 19.822, and P is the 10-year risk for that participant from above

- Female heart age = exp^((ln(ln(1– P)/ln(0.961))– K.woman)/ln(420.2))^

where K.woman= ln(7.57)*1.045 – 27.35, and P is the 10-year risk for that participant from above

**Comparator genetic risk**

- Men: P = 1 – 0.886^exp(ln(77.8)*ln(age) – ln(1.26)*0.9 – ln(77.8)*4.137)^
- Women: P = 1 – 0.961^exp(ln(442.4)*ln(age)^ ^– ln(1.11)*0.9^ ^– ln(442.4)*4.132)^
